# Trace Elements Analysis of Tunisian and European Extra Virgin Olive Oils by ICP-MS and Chemometrics for Geographical Discrimination

**DOI:** 10.3390/foods11010082

**Published:** 2021-12-29

**Authors:** Emna G. Nasr, Ekaterina N. Epova, Alberto de Diego, Radhia Souissi, Mohamed Hammami, Houyem Abderrazak, Olivier F. X. Donard

**Affiliations:** 1 Institut des Sciences Analytiques et de Physicochimie Pour l’Environnement et les Matériaux, Université de Pau et des Pays de l’Adour, 64000 Pau, France; emna.nsr@gmail.com; 2 Laboratoire des Matériaux Utiles, Institut National de Recherche et d’Analyse Physicochimique Technopole de Sidi Thabet, Ariana 2020, Tunisia; souissiradhia@yahoo.fr (R.S.); Mohamed.Hammami@inrap.rnrt.tn (M.H.); houyem.snani@yahoo.fr (H.A.); 3 Faculty of Sciences, Farhat Hached University Campus, University of Tunis El Manar, Tunis 1068, Tunisia; 4 Advanced Isotopic Analysis, Hélioparc, 64000 Pau, France; ekaterina.epova@ai-analysis.com; 5 Department of Analytical Chemistry, Faculty of Science and Technology, University of the Basque Country, 48080 Bilbao, Spain; alberto.dediego@ehu.eus

**Keywords:** olive oil, inductively plasma mass spectrometry, soil, trace elements, principal component analysis, geographical discrimination

## Abstract

The aim of this study was to investigate the levels of trace elements in olive oils from different locations and their use for geographical authentication. Concentrations of seventeen elements were determined in a total of 42 olive oils from Tunisia, Spain (Basque country), and southern France, and in nine soil samples from Tunisia by quadrupole inductively plasma mass spectrometry. The compilation of appropriate techniques integrated into the analytical procedure achieved a precision (RSD) between 2% and 15% and low limits of detection (between 0.0002 and 0.313 µg kg^−1^). The accuracy of the analytical method applied for olive oil analysis was evaluated using SRM NIST 2387 Peanut butter. The recoveries obtained after microwave-assisted digestion for the certified elements ranged between 86% and 102%. Concentrations of non-certified elements (V, Cr, Co, Ni, Ba, Rb, Sr, Cd, Pb, and As) were presented. The use of Pearson correlation applied on paired Tunisian oil/soil samples has shown that several elements (Mg, Mn, Ni, and Sr) were significantly correlated. The multivariate statistics using principal component analysis have successfully discriminated against three studied origins. The most significant variables were the elemental concentrations of Cu, Cr, Fe, Mn, Sr, V, and Zn. This study shows the potential of applying trace elements profiles for olive oil geographical discrimination.

## 1. Introduction

Olive oil is a natural food product largely consumed throughout the world. Its exceptional taste, its innumerable health benefits, and its nutritional value have made it a widely appreciated product [1,2]. The countries of the circum-Mediterranean basin, which are characterized by a climate favorable to olive growing, are the largest producers and exporters of olive oil, mainly Italy, Spain, Tunisia, and Greece. In Tunisia, in particular, the olive oil production sector represents a major economic resource for the country and is a strategic axis in its policy towards exportation, mainly towards the European community. Indeed, more than 75% of its olive oil production is mainly oriented towards exportation [3]. In recent years, Tunisian olive oils have been exported more and more in packaged bottles. Its policy is oriented towards the enhancement of this product and improving competitiveness in the international market. As a start, Tunisia has recently obtained a quality label, “The Appellation d’Origine Contrôlée” (AOC) olive oil of Teboursouk (3 March 2020, records of the World Intellectual Property Organization (WIPO)). Nevertheless, the proportion of oil packaged for export remains low, and Tunisia is hoping to double its packaged oil exports in the next five years applying the Hazard Analysis and Critical Control Point (HACCP) management system [4].

Tunisian olive oil exports are largely destined for the European Union; 97% of Spain’s extra-EU import of olive oil and 91% of France’s are Tunisian (International Olive Council, IOC). Therefore, ensuring the authenticity of olive oil is of general concern. The exports are mainly sent out in bulk (98%), opening the prospect of fraudulent practices [5]. Indeed, olive oil is now one of the most counterfeited food products [6,7]. This seemed to prompt the country’s awareness of the danger that threatens one of its flagship products. Between the early 90s and the year 2018, the number of articles that deal with the varietal or geographical origin of olive oil has increased exponentially, going from a few dozen articles to over 240 papers [8]. This increase is explained by the growing interest in this topical issue and also by the considerable development in the analytical field, allowing access to high precision and sensitive instruments. The analytical techniques used for the detection of oil adulteration are diverse. Besides organic oil components determination, trace elements analysis associated with chemometrics was successfully employed for oil geographical traceability issues.

In general, trace elements represent a good geographical tracer as they are naturally present in the soil at variable concentrations. They are absorbed through the roots and transferred to the aerial parts of the plant by translocation [9]. Their distribution in the final product then reflects the elemental signature of the soil of origin. Limited interest has been given to the influence of other factors interacting with the plant or with the final product, such as the extraction process of the olive oil or agricultural practices [10,11]. However, elements such as chromium, cadmium, and lead can be incorporated into the oil during its extraction, reflecting the manufacturing and packaging process [12].

Geographical authentication studies carried out on olive oil are gradually increasing but remain limited. Indeed, olive oil is a complex lipid matrix, especially with regard to its introduction into plasma-based instruments. It is characterized by a high organic content that requires advanced conditions to destroy the matrix, and incomplete mineralization leads to inaccurate concentration determination, interferences and can induce plasma extinction. Furthermore, trace elements are present in olive oil at low concentrations, sometimes below the limits of detection [13,14]. The detection technique most often used is inductively coupled plasma mass spectrometry (ICP-MS) due to its high sensitivity [15,16,17,18,19], and to a lesser extent, inductively coupled plasma atomic emission spectroscopy (ICP-OES) [20,21], graphite furnace atomic absorption spectroscopy (ETAAS/GFAAS) [22,23], and flame atomic absorption spectroscopy (FAAS) [24] are employed.

Most of the sample preparation techniques used are based on destructive methods of organic matter prior to spectroscopic analysis. Mineralization in a microwave oven has been the most used due to the ease of implementation, high recoveries, and destructive efficiency of this apparatus [17,25,26,27,28,29]. Several extraction methods were also developed based on the affinity of the elements with the extraction phase without having to destroy the organic matter [15,24,30,31,32]. Nevertheless, the recovery percentages of the extraction methods remain limited due to the complex organic structure of olive oil from which metals are difficult to extract. Other studies have adopted emulsion techniques as an alternative, but these are difficult to implement, especially in terms of maintaining the stability of the emulsion [21,33].

The application of multivariate statistical methods, mainly principal components analysis (PCA) and linear discriminant analysis (LDA) applied on the mineral content allowed the discrimination between food products and raw materials at different production lines [34], from different types of plants [35] and from different geographical origins [36]. In particular, olive oils were classified with variable resolutions according to their origin on the basis of several elements contents [25,26,37].

In the present study, we describe a method for accurate and precise trace element concentration determination in olive oils from Tunisia, France (southern France), and Spain (region of Basque country). To the best of our knowledge, very limited studies have studied the multi-elemental composition of Tunisian olive oil so far, and these studies focused on specific and limited geographical areas in Tunisia [10,26,38]. While it is characterized by varied and heterogeneous geology, the soil composition must vary significantly in different regions. For this reason, we have set as a first objective a sampling strategy that covers various olive oil-producing locations across the Tunisian geography, even the lowest productions for local consumption. For the samples preparation, we performed an innovative approach based on a multi-stage sample preparation procedure that allowed the destruction of the organic matter contained in olive oil in order to facilitate the analytes’ introduction into the ICP-MS. The quality control of the applied analytical procedure was performed using a standard reference material (SRM) NIST 2387 peanut butter. The recoveries were calculated for the concentrations of the certified elements; the non-certified elements were quantified with high precision and exclusively presented in this paper. The results obtained in this study will allow the creation of a database for the multi-elemental profile of Tunisian olive oils that can be useful for the quality assessment and the geographical characterization. The second objective of this work was to verify the correlation between the element concentrations in the soil and in the final product, which will allow the identification of the potential sources of elements that constitute the fingerprint of the geographical origin. Finally, the paper focused on the use of a common pattern recognition technique, the principal component analysis, as a tool for the classification of the samples according to their geographical origin.

## 2. Materials and Methods

### 2.1. Samples Collection and Preparation

#### 2.1.1. Samples from Tunisia

**Figure 1:**
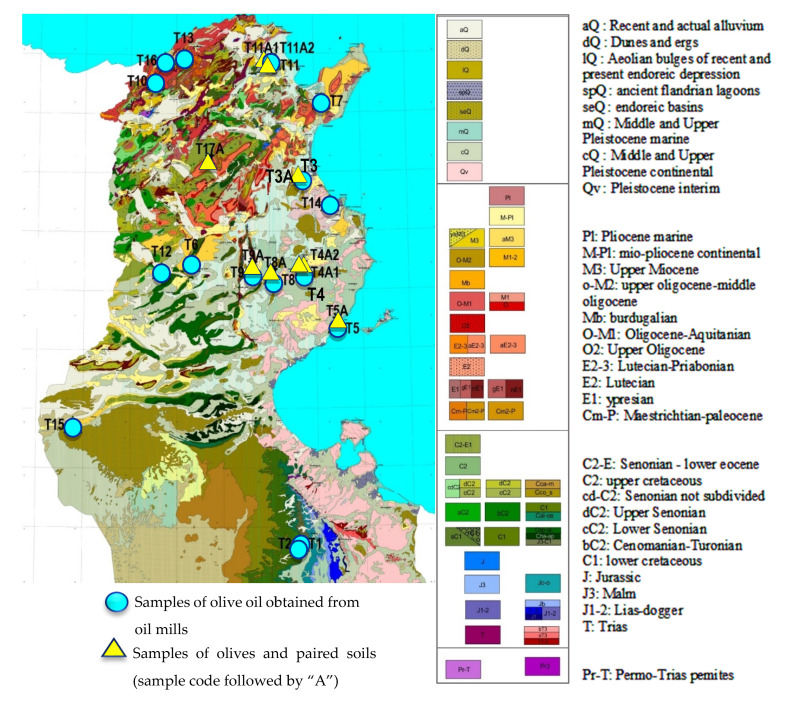
Sampling locations on Tunisian geological map.

*Olive oil sampling:* An extensive sampling campaign was conducted during the olive harvest period between 2019 and 2020 in thirteen Tunisian governorates. A total of 25 olive oil samples were collected as the following: sixteen packaged olive oils produced in oil mills were obtained from local producers based in different regions in Tunisia, and nine olive oils were extracted from olives collected in different olive orchards grown on different soils. Olive oil extraction was performed at the laboratory of the Olive Tree Institute of Tunisia (OTIT). Sampling locations are plotted on the geological map of Tunisia shown in Figure 1.

*Olive oil preparation:* For the extra virgin olive oil (EVOO) samples extracted from the olives collected, the following procedure was performed. About 4 to 6 kg of olives were handpicked and then kept in the refrigerator for a maximum of 24 h before the oil was extracted. The olive oil extraction method employed was the ABENCOR method (https://sistemaabencor.com/en/, accessed on 25 November 2021). It includes leaf stripping and washing of the olives. Then, the grinding of the olives was performed in a hammer mill in order to obtain an olive paste. The resulting olive paste was mixed for 45 min in a mixer. During this step, a small volume of distilled water was added in order to facilitate the oil droplets released from the paste.

During the mixing step, the temperature was controlled not to exceed 27 °C. The final step was centrifugation. The olive paste obtained was introduced in a centrifuge system at 3500 rpm for 1 min. This allowed the separation of the dry pomace from olive oil, which was mixed with the wastewater. The olive oil was then separated from the water by natural decantation. All metallic surfaces in direct contact with olives, paste, and olive oil were made of stainless steel and were carefully washed after each sample run. The obtained olive oils were carefully transferred in brown glass bottles that had been previously washed with distilled water and then stored away from heat and light.

Soil sampling: Under each sampled tree from the selected orchards, paired soils were collected for multi-elemental analysis as well. A total of 9 soil samples were collected at a depth of 60 cm using a pickaxe. This depth was selected since it was deep enough to avoid soil with surface treatment procedures. Once collected, the soil samples were air-dried for a few days and sieved through a 2 mm sieve. Then, 50 g were taken from each sample by the quartering method.

#### 2.1.2. Samples from Europe

Apart from Tunisian olive oils, EVOOs from European locations were obtained and analyzed to compare their multi-elemental profile with that of the Tunisian ones. Seven olive oil samples were obtained from different locations of the Spanish Basque Country: Moreda, Lantziego, and Oion (province of Araba), and Añorbe, Mendavia, Cintruenigo, and Arroniz (province of Navarre). Ten protected designation of origin (AOP) EVOOs from southern France were also obtained from supermarkets (AOP Provence, AOP Nîmes, AOP Nice, and AOP Nyons).

Table 1 summarizes the olive oil and soil samples’ geographical origins as well as the number of samples. Information about the geographical location, cultivar, geological formation, and applied agricultural practices are presented in Table 2.

### 2.2. Multielemental Analysis

#### 2.2.1. Reagents and Chemicals

Sub-boiled nitric acid (69% Instra analyzed JT BAKER), hydrogen peroxide (30–32% Optima, Fisher scientific, Illkirch, France), and Ammonium acetate (>98%, Sigma Aldrich, Darmstadt, Germany) was used for samples preparation. All solutions were diluted using ultrapure water (Milli-Q system, resistivity 18.2 MΩ cm, Millipore, Burlington, MA, USA). Multi-elemental standard solutions CCS-4 and CCS-6 (Inorganic ventures, Christiansburg, VA, USA) were used to build the external calibration curve. Mono-elemental solutions of Y, Rh, and Ir were used as internal standards for ICP-MS measurements.

#### 2.2.2. Olive Oil Mineralization

**Figure 2:**
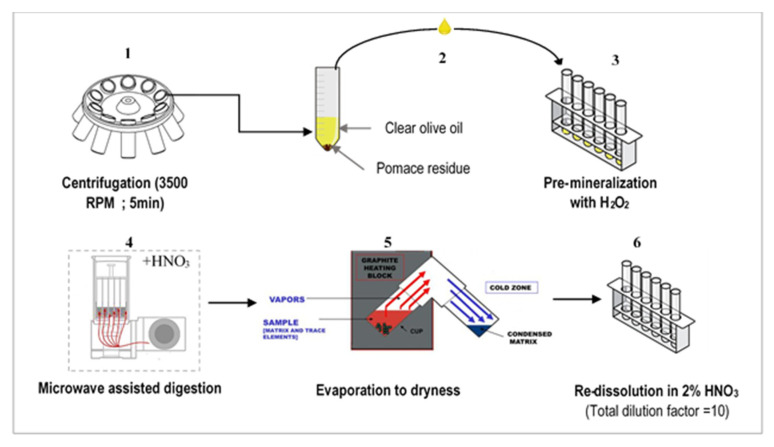
The analytical procedure for olive oil preparation prior to ICP-MS analysis: (1) Centrifugation; (2) Clear olive oil recovering; (3) Pre-digestion with H_2_O_2_; (4) Microwave assisted digestion; (5) Evaporation to dryness; (6) Re-dissolution in 2% HNO_3_.

Prior to ICP-MS analysis, a multi-stage procedure was performed (Figure 2). First, the organic matter contained in olive oils was destroyed through mineralization. Then, the obtained mineralized solutions were subjected to evaporation to dryness, followed by dissolution in the minimum volume required for analysis. Prior to the mineralization, the olive oil samples were centrifuged at 3500 rpm for 5 min using a ROTOFIX32A (HETTICH, Atlantic Labo, Bruges, France) centrifuge in order to eliminate any pomace residue according to previous recommendations [16]. Then a mass of 0.5 g of olive oil from the upper oil totally clear and not containing any pomace residue was carefully transferred to quartz tubes previously cleaned following a strict cleaning protocol as follows: the digestion tubes containing 50% nitric acid were placed in a microwave system (Ultrawave SRC technology, Milestones, Sorisole (BG), Itlay) with a specific cleaning program. Then, the tubes were washed in 10% *v*/*v* HNO_3_ and in 10% HCl successively and finally rinsed with ultrapure water.

For the samples digestion protocol, 0.5 mL of 30% hydrogen peroxide was added to 0.5 g of the olive oil to be mineralized. The mixture was left overnight for pre-mineralization at room temperature. The next day, 5 mL of sub-boiled nitric acid (69%, Instra Analysed Reagent, J.T.Baker, Fisher Scientific, France) was gradually added since olive oil is a highly reactive matrix. The final mixture was then digested in a microwave system (Ultrawave SRC technology, Milestones, Sorisole (BG), Italy) following an optimized gradually increasing heating program up to 250 °C where the temperature was maintained for 20 min (P max = 110 bar). The resulting clear solutions were then transferred to clean PFA vials (Savillex Corporation, Eden Prairie, MN, USA) and evaporated to dryness in a closed-medium sample evaporation apparatus (Evapoclean 25 mL, Analab, 67,800 hoenheim, France). The dry residue was finally re-dissolved in 5 mL of 2% sub-boiled nitric acid prior to analysis. The total dilution factor obtained was then equal to ten.

### 2.3. Soil Extraction

Prior to ICP-MS analysis of the soil samples, only elements in the exchangeable fraction of soil were extracted. A large array of analytical methods involving extraction procedures has been published and extended, following the integrative work performed with the former BCR three-step sequential extraction procedure on the validation of sequential extraction techniques [39,40,41]. In this work, the soil extraction procedure was performed following closely the method proposed by Ure et al. (2006) based on ammonium acetate extracts [42]. Indeed, ammonium acetate is largely employed in soil extraction techniques as it is known for its cation exchange capacity, exchangeable bases, and plant-available nutrients extraction. Briefly, a volume of 3 mL of 1 M ammonium acetate at pH 7 was added to 2 g of each soil sample to release elements in exchangeable fraction. The mixture was shaken for 16 h on a shaking plate (Edmund Buhler Shaker, D-72411 Bodelshausen, Germany) then centrifuged at 3500 rpm for 5 min. The supernatant aqueous phase was then carefully transferred using a pipette then diluted in 2% sub-boiled nitric acid prior to analysis by ICP-MS.

### 2.4. Multielemental Analysis 

The multi-elemental analysis of the obtained extracts from soils and oils was performed using an ICP-MS Plasma Quant Elite spectrometer (Analytik Jena, 07745 Jena, Germany). All samples were analyzed for a total concentration of 17 elements: As, Ba, Ca, Cd, Co, Cr, Cu, Fe, K, Mg, Mn, Ni, Rb, Sr, Pb, V, and Zn. The instrumental operating conditions and measured isotopes are presented in Table S1 (Supplementary Materials). The analysis was performed using two operating modes: The standard mode where no gas was supplied into the cell, and the integrated collisional reaction cell (iCRC) in the reaction mode where He and H were used as collision gases into the cell, and the iCRC mode was applied in order to reduce spectral interferences, including polyatomic interferences. The list of isotopes detected for each operating mode is detailed in Table S1 (Supplementary Materials).

The calibration solutions at eight different concentrations ranging from 0.01 μg L^−1^ to 500 μg L^−1^ were prepared by appropriate dilution of multi-elemental standard solutions CCS-4 and CCS-6. Yttrium (Y), rhodium (Rh), and iridium (Ir) at a concentration of 2.5 μg L^−1^ were used as internal standards for the low, medium, and heavy masses, respectively, in order to correct instrumental drifts. Samples concentrations were calculated after applying the corrections with the blank, the internal standard, and the analytical blank. First, the calibration blank (2% HNO_3_ spiked with the internal standard) signal was subtracted in order to ensure that the ICP-MS measurement provides a zero signal when no analyte is present. Then, the instrumental sensitivity drifts were corrected by the internal standard signal. Finally, an “analytical blank” (69% HNO_3_, H_2_O_2_) prepared using the whole set of reagents was mineralized and underwent the same preparation procedure as the samples under the same conditions. This blank reflects the potential contamination that occurred during the whole samples preparation protocol, and it was subtracted from the samples’ elemental concentrations in order to calculate the “true” concentration of each element.

All labware was washed in 10% *v*/*v* HNO_3_ and rinsed with ultra-pure water before use.

### 2.5. Analytical Quality Control 

The standard reference material NIST SRM 1643f (trace elements in water, National Institute of Standards and Technology, Gaithersburg, MD, USA) was used for quality control of the instrumental measurements. The NIST SRM 2387 (peanut butter, National Institute of Standards and Technology, Gaithersburg, MD, USA) was used to evaluate the accuracy of olive oil mineralization through recoveries of the certified elements. NIST SRM 2387 peanut butter was chosen since a natural vegetable oil certified for trace elements concentrations is not available. Despite the fact that this matrix is not totally equivalent to olive oil composition and viscosity, the chemical composition of this fatty acid-rich matrix is very close to that of olive oil. For the non-certified elements (As, Ba, Cd, Co, Cr, Ni, Pb, Rb, Sr, and V), a series of microwave-assisted mineralization were performed on NIST SRM 2387 prior to ICP-MS analysis. The average concentrations for inter-day and intra-day results obtained for these elements on the basis of 15 replicates are presented in Table 3.

In every mineralization run, a blank and a triplicate of NIST SRM 2387 were digested and then analyzed. Recoveries for six certified elements concentrations (Cu, Fe, K, Mg, Mn, and Zn) in NIST SRM 2387 were calculated as the ratio: (Measured concentration/Certified concentration) * 100. The recoveries (R (%)) obtained after microwave-assisted digestion were satisfactory, ranging between 86% and 102% (Table 3).

The limits of detection (LODs) and limits of quantification (LOQs) were calculated as three times the standard deviation (SD) and ten times of SD on the basis of 10 blanks, respectively. The obtained LODs and LOQs were significantly low (Table S2: Supplementary Materials): LODs were between 0.0002 and 0.313 µg kg^−1^ and LOQs between 0.0007 and 1.042 µg kg^−1^. Except for Cd in olive oil, all the measured concentrations were greater than LOD and LOQ. Linearity was satisfactory; R^2^ was above 0.9995 for all analyzed elements. The precision was evaluated through the relative standard deviation (RSD) and calculated on the basis of olive oil triplicates. The RSDs were satisfactory; the values obtained were low, ranging between 1% and 14% (Table S2: Supplementary Materials).

### 2.6. Statistical Data Analysis

For the classification of olive oil samples according to their geographical origin (Tunisia, Spain, and France), a multivariate analysis was performed using SIMCA V16.0.1. The Principal Components Analysis (PCA) is an unsupervised method aiming to find new variables called dimensions calculated from a covariance matrix of the original variables while preserving as much as possible the statistical information (variability), allowing summary and visualization of the information in large datasets. The samples classification was accomplished by the PCA score plot, and the determination of the most discriminating elements was conducted by the loadings plot. Spearman’s correlation was calculated for the trace element concentrations in an exchangeable fraction of soil and corresponding olive oils in order to verify the possible correlation between both. Spearman’s correlation factors were calculated using OriginLab 2018.

## 3. Results and Discussion

### 3.1. Trace Elements Concentrations in Olive Oils

The trace elements determined in the olive oils analyzed presented a wide range of concentrations ranging from less than 1 µg·kg^−1^ up to the range of mg kg^−1^. Table 4 presents the median and the ranges of concentration of 17 elements in olive oil samples classified according to their geographical origin (Tunisia, Spain, and France). They were compared to the concentrations reported in the literature. In general, except for Fe, all the elements analyzed displayed slight but noticeable levels of concentration in all the olive oil samples. The first group of elements includes those displaying the lowest concentration ranges. They are As, Cd, Co, Pb, and Rb. These elements’ median concentrations were less than 1 µg kg^−1^. They are the most difficult to detect in a complex matrix such as olive oil due to the high organic load and viscosity for introduction in the ICP-MS with typical nebulization.

*Arsenic*: The concentration of As obtained in Tunisian, Spanish, and French olive oils analyzed were low, around 0.1 µg kg^−1^, and were notably lower than those reported in the literature. Indeed, the concentrations of As recorded varied between 0.05 µg kg^−1^ and 0.16 µg kg^−1^ in European oils and were between 0.06 µg kg^−1^ and 0.88 µg kg^−1^ in Tunisian oils. These results were mostly in agreement with the concentrations previously reported in Spanish olive oils [43] and were notably lower compared to other studies conducted on oils from Tunisia and Italy [26,28].

*Cadmium*: The values obtained for Cd were below the LOD in all olive oil samples. Cadmium is one of the major contaminants that can expose humans to health risks. It can be absorbed by plants from the soil and then translocated to the edible parts of the plant, and it can also be released to edible oils stored in tanks and plastic packaging (Ismael et al., 2019). In most of the published studies, cadmium was either not analyzed or was not detected in olive oil [31,44]. Otherwise, when quantified, concentrations of Cd varied between 0.001 µg kg^−1^ and 0.15 µg kg^−1^ [16,21,45,46].

*Cobalt*: The concentration levels of Co in Tunisian olive oils ranged from 0.03 µg kg^−1^ to 0.31 µg kg^−1^. The concentrations were similar to those reported for European oils, ranging between 0.04 µg kg^−1^ and 0.17 µg kg^−1^. These concentrations were in full agreement with the concentrations previously evaluated in Spanish and Italian olive oils [25,28,29].

*Lead*: The concentrations of Pb were 10 times higher than those recorded for Co but were as well at low levels. In general, in the European oils, the concentrations of Pb did not exceed 1.35 µg kg^−1^. These values were in agreement with those previously reported results from Spain, Portugal, and France [15]. Slightly higher values were found in oils from Tunisia. The highest value was equal to 2.16 µg kg^−1^, and this was found in olive oil from Jendouba (T16) sampled near a Pb-Zn mining deposit (Table 2). Lead is a hazardous contaminant that can be assimilated by plants and is then accumulated at variable concentrations depending on the location of the Pb emission source [47]. These relatively high values of Pb in the Tunisian oils were in agreement with recent results from oils originating from four geographic locations (Monastir, Medenine, Gafsa, and Sfax). They also displayed relatively high concentration levels, between 5 µg kg^−1^ and 7.4 µg kg^−1^ [26]. In this same study, concentrations of Rb and Sr were also investigated in olive oils.

**Table 4:** Median values and ranges of concentrations (µg kg^−1^) of trace elements in olive oil samples from Tunisia, Spain (Basque country), and southern France, and ranges of concentrations reported in the literature.

Element	Tunisia		Spain (Basque Country)		France		Literature *
Cd	<LOQ		<LOQ		<LOQ		(0.001–0.15)
Co	0.09	(0.03–0.31)	0.07	(0.04–0.10)	0.07	(0.04–0.17)	(0.023–11)
As	0.14	(0.06–0.88)	0.10	(0.09–0.14)	0.08	(0.05–0.16)	(0.2–26.6)
Rb	0.35	(0.09–1.85)	0.55	(0.28–0.95)	0.66	(0.35–1.94)	(0.036–2.6)
Pb	0.88	(0.57–2.16)	0.53	(0.28–0.94)	0.70	(0.47–1.35)	(0.18–6.40)
Ba	1.21	(0.45–5.17)	1.28	(0.93–2.30)	1.61	(0.92–17.6)	(0.31–12.3)
Sr	2.58	(1.18–5.04)	1.73	(1.21–2.11)	2.50	(1.00–3.55)	(1.52–48.9)
V	3.25	(2.10–5.45)	1.14	(0.52–1.56)	0.77	(0.12–1.53)	(4.2–5.8)
Mn	5.03	(3.58–17.3)	1.57	(0.96–2.70)	2.34	(1.08–3.71)	(4.4–40)
Ni	3.50	(2.02–11.6)	2.65	(1.86–3.44)	3.88	(2.22–6.88)	(5.95–173)
Cu	8.74	(3.62–23.5)	5.26	(3.10–11.1)	6.16	(4.40–6.55)	(3.35–66.4)
Cr	10.3	(7.11–16.8)	8.48	(3.45–11.2)	14.4	(11.9–18.2)	(15.4–437)
Zn	98	(39–195)	129	(100–152)	106	(33–138)	(7–290)
Fe	1240	(169–1310)	102	(80.7–117)	129	(54.7–190)	(67.5–1610)
Mg	208	(138–582)	226	(91.9–488)	368	(160–785)	(223–1200)
K	534	(128–3740)	601	(214–2970)	527	(142–1530)	(498–98,000)
Ca	1240	(610–2280)	942	(607–1990)	1080	(580–1640)	(76–10,790)

* The literature data was collected from the previously reported results [10,15,16,18,26,27,28,30,31,33,38,43,44,48,49,50,51].

*Rubidium and Strontium*: The rubidium reported concentrations ranged between 2.5 µg kg^−1^ and 3.5 µg kg^−1^ and were in agreement with the concentrations found in the present study. However, the reported concentrations of Sr were relatively high, ranging between 33 µg kg^−1^ and 37 µg kg^−1^, which is up to 30 times greater than the concentrations found in this study. Both Rb and Sr concentrations varied significantly in Tunisian oils.

The concentrations of Rb ranged between 0.09 µg kg^−1^ and 1.85 µg kg^−1^ and concentrations of Sr between 1.18 µg kg^−1^ and 5.04 µg kg^−1^. This variability is related to the varied geology of Tunisian soil. Indeed, Rb and Sr contents are strongly linked to the geochemical composition of the soil of origin [52]. This variation was less significant in the Spanish and French olive oil samples. Indeed, compared to the large geographical distribution of sampling performed in Tunisia, the samples from the Spanish Basque country and France were obtained from a limited and restricted geographical area.

The second group of elements includes the elements occurring at medium concentrations, ranging from a few µg kg^−1^ up to 100 µg kg^−1^. It includes Ba, Cr, Cu, Mn, Ni, Sr, V, and Zn.

*Barium*: Ba is naturally present in the environment and is a non-essential element for plant growth. The high concentrations of Ba in soil could be related to the geological formation and also to contamination through industrial activities (paints, ceramics, and glass) [53]. Ba concentrations ranged from 0.45 µg kg^−1^ to 5.17 µg kg^−1^ in Tunisian oils. These values were lower than those reported by Damak et al., (2019) [26]. Similar median concentrations were obtained in Spanish and French oils, respectively equal to 1.28 µg kg^−1^ and 1.61 µg kg^−1^. An outlier was recorded in the sample originating from Nîmes, the concentration of Ba was equal to 17.6 µg kg^−1^. Such high barium concentrations had been previously recorded in Spanish olive oils [29,33].

*Chromium*: Cr is also non-essential in plant growth and development but is assimilated by roots with other essential elements [54]. Concentrations of Cr in Tunisian olive oils varied between 7.11 µg kg^−1^ and 16.8 µg kg^−1^. Similar levels of Cr were found in the other European oils analyzed and were all in line with the previously reported results from Spain [50]. A wide range of concentrations was reported in the literature for Cr, from a few tens of µg kg^−1^ levels up to hundreds of µg kg^−1^ [21,25,28,50,51].

*Copper*: Concentrations of Cu varied between 3.62 µg kg^−1^ and 23.5 µg kg^−1^ in the Tunisian oil and were significantly greater than the values reported in the literature [38]. In the European oils, the concentrations of Cu ranged between 0.04 µg kg^−1^ and 0.17 µg kg^−1^ and were significantly low compared to the previous findings in Italy, Spain, and Turkey [15,18,21,43,50,55]. The presence of Cu in the soils is necessarily related to the natural geogenic background levels but could be altered by different cultivation processes and, therefore, could be found at a wide range of concentrations.

*Vanadium and Manganese*: Both V and Mn are essential nutrients for plant growth and development at low concentrations. Although there are sources of vanadium contamination, such as metallurgical industry and mining activities, its composition in soil mainly reflects that of the rocks [56,57]. In previous studies, vanadium was rarely analyzed in olive oil and hardly detected due to its very low concentration [33]. In the present study, the lowest value obtained was equal to 0.12 µg kg^−1^ and was 10 times higher than the LOD. The concentrations of V in Tunisian olive oils ranged from 2.1 µg kg^−1^ to 5.45 µg kg^−1^. Mn concentrations ranged from 3.58 µg kg^−1^ to 17.2 µg kg^−1^. Both ranges of concentrations were in agreement with the previously reported results for Tunisian oils [26]. Lower concentrations were found in the Spanish and the French olive oils since the concentrations detected fluctuated between 0.12 µg kg^−1^ and 1.56 µg kg^−1^ for V and did not exceed 3.71 µg kg^−1^ for Mn. Both levels detected were in agreement with the previous results reported in European oils [15,31].

*Nickel*: Median concentrations of Ni were similar in olive oils from the three different origins ranging between 2.65 µg kg^−1^ and 3.83 µg kg^−1^. Very limited information is available for levels of Ni in the Tunisian olive oils; we can only refer that the values obtained from trees irrigated with treated wastewater presented higher levels of Ni [10]. Other published results from Spain and Italy also showed high concentrations ranging between 10 µg kg^−1^ and 60 µg kg^−1^ [28,50]. In general, the presence of Ni in the soil is mainly related to the parent rock composition; however, it can be accumulated in the plants as a result of agricultural and industrial practices, and therefore, nickel would be found in a wide range of concentrations in soil and similarly in olive oil [58].

*Zinc*: Concentrations of Zn varied between 39 µg kg^−1^ and 195 µg kg^−1^ in Tunisian oils. The highest values were recorded in the sample T16, sampled close to a Zn mining site. Thus, the high concentrations of Zn in olive oil can be related to the direct uptake of Zn from the polluted soil or to the indirect intake from dust Zn deposited on the leaves that are translocated into the olive fruits [59]. Similar ranges were obtained in European oils, ranging from 33 µg kg^−1^ to 152 µg kg^−1^. These results agreed most with reported values in olive oils from Trentino, Italy [15] and from Tunisia [26]. Notably, higher average concentrations were recently recorded in European olive oils, up to 492 µg kg^−1^ [18].

The third group of elements includes the elements occurring at relatively high concentrations from a few hundred µg kg^−1^ to the range of mg kg^−1^. It involves Ca, Fe, K, and Mg. They are all necessary for plant growth and development. 

*Calcium and Potassium*: Concentrations of Ca and K in Tunisian olive oils were in agreement with those obtained from olive trees irrigated with treated wastewater [10]. This could be related to the source of irrigation water of our sampling locations. For the European samples, concentrations of K were high in some samples from the Spanish Basque country compared to French oils. This could be due to the amendment of olive trees in Basque locations with NPK (Table 2). The concentrations of Ca in European olive oils were found in comparable concentration ranges, between 580 µg kg^−1^ and 1990 µg kg^−1^. These values were lower than those previously reported in Spain [29].

*Iron*: Concentrations of Fe in Tunisian samples ranged from 169 µg kg^−1^ to 1310 µg kg^−1^. The median value was equal to 1248 µg kg^−1^. The highest values were obtained in olive oils produced at OTIT using the Abencor extraction method. This relatively high concentration of Fe was likely to be related to direct contamination by the extraction process of the olive oil. Indeed, Zeiner et al., (2010) pointed out that the high metal content in olive oils could be related to the production process [60]. Levels of Fe were much lower in European oils, ranging from 54.7 µg kg^−1^ to 160 µg kg^−1^. These results were more in agreement with the previously reported concentrations [18,28,50].

*Magnesium:* The concentrations of Mg in olive oils from Tunisia, ranging from 138 µg kg^−1^ to 582 µg kg^−1^, were lower than those previously reported [26]. Elemental Mg content in European olive oils was in contrast similar to concentrations reported in different Spanish locations [29].

This wide range of concentrations and the inter/intra-country variability is certainly related to soil geochemistry and the possible contribution from cultural processes or anthropogenic activities.

### 3.2. Relationship between Olive Oil and Soil Elemental Content in Tunisia

The multi-elemental profile of the soil samples was determined, and the median, as well as the range of elements concentrations, are presented in Table S3 (Supplementary Materials). In order to evaluate the possible correlations between bioavailable elements contents in soil and elements concentrations recorded in the paired olive oils, we performed a correlation test. Pearson correlation coefficient was calculated for the soil exchangeable fraction extracts and corresponding olive oil elemental composition.

The correlation coefficients were evaluated in nine Tunisian sampling locations (T3A, T4A, T4A’, T5A, T8A, T9A, T11A, T11A’, and T17A), presenting different soils characteristics. The results obtained are presented in Table 5 and highlight that, despite the limited number of samples, only four elements (Mn, Ni, Mg, and Sr) out of seventeen presented a statistically significant correlation between soil extracts and olive oil elemental content (*p* < 0.05).

**Figure 3:**
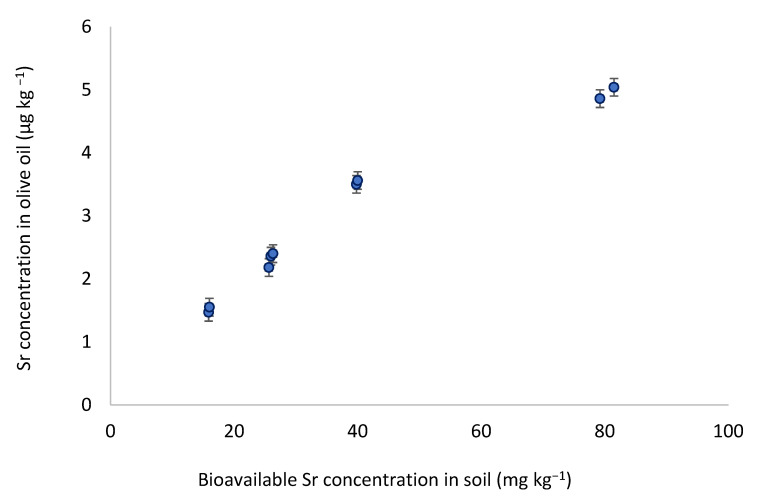
Linear correlation between Sr content in olive oil and in bioavailable Sr of soil extracts.

Both Mg and Sr correlation coefficients, respectively equal to 0.78 and 0.85, indicated a high positive relationship between their concentration in soil and in olive oil. Figure 3 displayed the strong correlation between the bioavailable Sr content in the soils and its concentration in olive oils. The correlation coefficient for Mn (*r* = 0.63) indicated a positive correlation. It has been shown that a significant amount of Mn assimilated by the olive tree is retained in the olive leaves [61], which could explain the moderate correlation since olive oil is obtained from the olive fruit. The Ni correlation coefficient (*r* = −0.8) demonstrated a high negative correlation. This indicates that the higher the concentration of Ni in the soil, the less Ni is found in the olive oil. This might be related to the accumulation of Ni in different organs of the plant [62].

Elements such as As, Ba, Ca, Cd, Co, Cr, Cu, Fe, K, V, Zn, Pb, and Rb are first derived from geogenic sources, but most of them can also be originating from various anthropogenic activities either via agricultural practices or during the olive oil extraction process including storage. Their concentrations did not show any significant correlation between the soils and the resulting produced olive oils (*p* > 0.05).

The accumulation of Fe in the olive tree merits a special mention. In this study, we observed significantly high Fe contents in olive oils extracted in the OTIT applying the Abencor extraction method compared to those produced in an oil mill. These findings would suggest that the production process of olive oil could be a source of iron contamination. During the Abencor extraction, olive oil is in direct contact with metal surfaces made of stainless steel. Iron is the main constituent of stainless steel, and thus, it could be transferred to olive oil during the extraction process. This is also the case of chromium contents which would also indicate direct contamination during the olive oil extraction.

In general, elements contents in the soils and their assimilation by plants can be subjected to fluctuations according to the agro-climatic conditions [11]. Under specific environmental conditions, the uptake and accumulation of elements from the soil are affected. As a response to water stress, plants accumulate some elements in the roots that will be translocated later to satisfy their needs [63,64]. Furthermore, genetic determinism has been demonstrated to affect the accumulation of elements by the olive tree. Beltràn et al., (2015) demonstrated that there are differences in some elements concentrations (Ba, Cu, Rb, and Zn) between different olive cultivars [25]. Competitiveness between chemically similar elements during plant water uptake can also result in different levels of accumulation. For example, concentrations of Rb and K that exhibited a non-significant correlation between soil and olive oil are in competition for entry into the plant cell due to their chemical similarity [61]. When the correlation is not significant between the olive oil and soil composition, this cannot exclude the hypothesis that the correlation could be significant between the soil and other organs of the olive tree. It has been previously demonstrated that the translocation and distribution of assimilated elements in the olive tree are not homogenous, and elements would be accumulated in different parts of the plant (leaves, roots, and stem bark), in some cases more than in the fruit [62]. Therefore, elements distribution in olive oils is not only related to the sources of trace elements in the plant, whether natural or anthropogenic, but also determined by the intrinsic properties of the plant. Some elements such Mg, Mn, Ni, and Sr have preserved the elemental signature of the soil, and therefore, they are reliable for geographical traceability. Other elements that did not exhibit a significant correlation could also be used for discrimination of geographical locations with different environmental conditions.

### 3.3. Geographical Classification of Olive Oils

**Figure 4:**
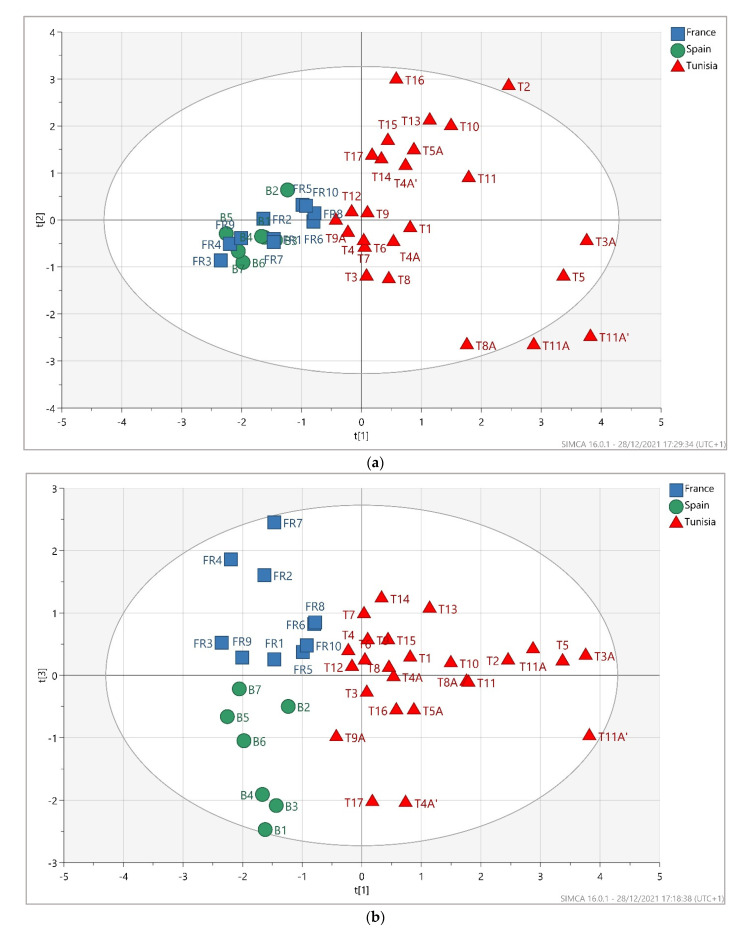

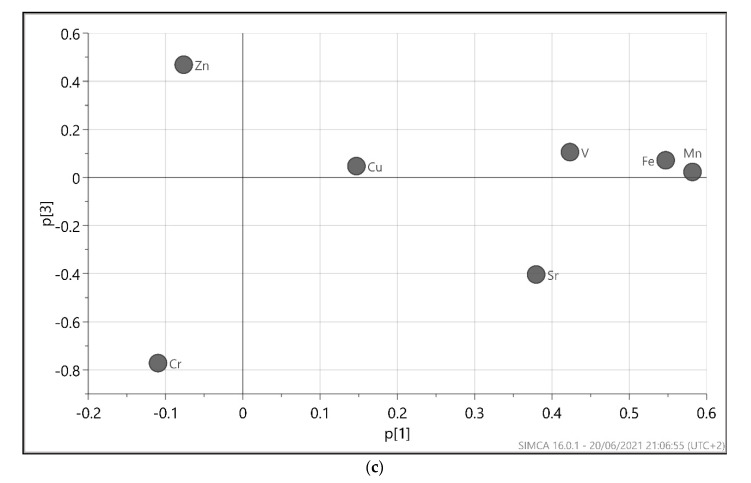
PCA score plot colored according to geographical origin (Tunisia, Spanish Basque country, and southern France): (**a**) PC1 vs. PC2; (**b**) PC1 vs. PC3; (**c**) loadings plot for PC1 and PC3.

The multi-elemental profile of the olive oils analyzed showed a slight but noticeable variability between the samples from different origins; however, it did not allow direct geographical discrimination based on their concentrations. In order to reduce such a large dataset without drastic loss of information, a principal component analysis (PCA) was applied. A series of PCAs were performed with different elements combinations. The elements at closely similar concentrations in all the olive oils did not contribute to the classification of the samples with respect to their geographical provenance. The most discriminating elements with notable loading factors that allowed the best separation of the samples were used as variables. The average concentrations of seven elements (Mn, Fe, V, Cr, Sr, Zn, and Cu) were used as variables to perform principal component analysis (PCA). Among these elements, we have demonstrated that only Mn and Sr contents in olive oil were correlated to that of soil. The first three PCs were extracted and explained together 71.58% of the variance. The first principal component (PC1) explained 34.6% of the variance, and the second principal component (PC2) retained 21.1% of the total data variance. The score plot PC1 vs. PC2 (Figure 4a) allowed establishing a separation between two groups of samples: Tunisian and European olive oils according to PC1. Even if Tunisian oils were produced two ways, the score plot did not show a separation according to the extraction process. Some outliers were observed for the Tunisian samples. T2, T3A, T5, T8A, T11A, and T11A’ had higher scores on PC1 compared to the cluster of samples from Tunisia. These samples are characterized by high concentrations of Fe compared to the median value, ranging between 4090 ± 21 µg kg^−1^ and 8310 ± 76 µg kg^−1^, and most of them were produced in OTIT (Abencor extraction). PC2 did not allow any separation between samples. The score plot PC1 vs. PC3 presented in Figure 4b was then performed and showed a clear clustering of olive oil samples according to their origin (Tunisia, Spain, and France). There is a clear separation between samples with high predictive accuracy (R^2^X(cum) = 0.69). The third principal component was effective for the separation of French and Spanish samples with 15.9% of the variation. Therefore, the PCA, which is an unsupervised method, allowed to separate olive oil samples from different geographical origins based on trace elements.

The loadings plot shown in Figure 4c can be used to identify the contribution of the elements to the samples classification as well as the correlations between variables. Fe and Mn are likely to be correlated. Indeed, both elements had a similar geochemical behavior [65]. They presented the highest loadings on PC1 (between 0.5 and 0.6). Therefore, they strongly influenced the first component that separates Tunisian and European olive oils. Concentrations of Fe and Mn exhibited the highest values in olive oil samples from Tunisia (Table 4). While concentrations of Mn in olive oil were found to be correlated to that of soil, Fe did not show a significant correlation. Therefore, the separation between samples from Tunisia and from Europe is not only related to the soil composition. Most likely, the olive oil extraction process has an important role in olive oils discrimination since Fe was highly loaded on PC1.

A slightly lower loading value was obtained for V on PC1, which was equal to 0.42. Vanadium content in olive oil was not correlated with soil. Thus, external sources of V pollution may be responsible for the significant differences in V content. The highest values were observed in Tunisian olive oils (Table 4).

Strontium exhibits similar absolute loading values on PC1 and PC3. Therefore, it enables the separation of all the samples according to their geographical provenance.

Copper moderately influences PC1 (0.15) and poorly influences PC3 (≈0). Furthermore, the Zn loading on PC1 (<−0.1) indicates that Zn weakly influences PC1, but it had a greater influence on PC3 with a large positive loading (0.47). Concentrations of Zn and Cu are not correlated with soil composition.

Chromium is highly loaded in PC3. Therefore, chromium content exhibits an important statistical weight for the discrimination of French and Spanish olive oils. As reported in Table 4, concentrations of Cr were higher in olive oils from France. The high discriminatory value for Cr could be related at first to the geogenic signatures of the respective areas or/and associated with the signature of the packaging and the olive oil extraction process.

Based on this exploratory chemometric analysis, it has been shown that trace elements content can be used for the geographical discrimination of olive oil. This separation is based on elements that are mainly related to the soil geochemical background and also on elements originating from anthropogenic activities and the production process.

## 4. Conclusions

The presented work is one of the large-scale studies performed on Tunisian olive oils in terms of representing the production regions. The quality and the protection of the olive oil sector are among the main economic priorities in Tunisia.

The findings of this study suggest that the multi-elemental composition of olive oil can be successfully used for the geographical discrimination issue. The accurate determination of 16 elements concentrations was performed with high precision in Tunisian, Spanish, and French olive oils using the quadrupole ICP-MS after microwave-assisted digestion. Under the optimal conditions of mineralization and analysis, relatively low values of LOD, LOQ were obtained. The RSD values obtained indicate a good precision. The SRM NIST 2387 peanut butter was used for quality control of the applied analytical procedure, analysis of the certified and non-certified elements was performed.

The trace elements levels showed a wide range of concentrations and were in agreement with the previously reported results. Among the analyzed elements, four out of seventeen elements (Mn, Sr, Mg, and Ni) were found to be strongly correlated to the bioavailable soil composition. This states the transferring of the geochemical signature of the soil to the olives and then to oils. On another side, the failure to identify clear correlations for the remaining thirteen elements undoubtedly indicates that the elemental composition of olive oil could be affected either by plant elements uptake and accumulation depending on agro-climatic conditions and genetic determinism or through the production process and storage conditions. The PCA classification of olive oils according to their origins using seven elemental concentrations (Cu, Cr, Fe, Mn, Sr, V, and Zn) allowed successful discrimination with high predictive accuracy not only between European and non-European origins but also between Tunisian, French, and Spanish origins. The attribution of olive oils to their origin was not hindered by the type of the production: whether manually extracted in a laboratory or pressed on a mill, the origin proved to have a more significant effect on the variation of multi-element concentrations in olive oils.

The results obtained in this study are promising and suggest a rapid and reliable method for the geographical discrimination of olive oil based on trace elements content. In future work, isotopic compositions of several elements (O, C, N, and Sr) will be investigated and applied with the aim of enhancing the provenance discriminating ability.

## Figures and Tables

**Table 1:** Extra virgin olive oil and soil samples: geographical origin and number of samples.

Geographical Origin	Tunisia			France (South)	Spain (Basque Country)
Samples	EVOO (produced in oil mill)	EVOO (produced in OTIT)	Soil (0–60 cm)	EVOO	EVOO
Number of samples	16	9	9	10	7

**Table 2:** Olive oil samples: geographical location, cultivar, geological formation, and applied agricultural practices.

Country of Origin (Number of Samples)	Geographical Location (Number of Samples)	Sample Code	Cultivar	Bedrock (Mineralization)	Agricultural Practices
Tunisia (*n* = 25)	Tataouine (*n* = 2)	T1	Zarrazi	limestones and marls	nt
		T2	Dhokkari		
	Sousse (*n* = 2)	T3	Chemlali	calcareous and gypsum crusts	nt
		T3A	Chemlali		
	Mahdia (*n* = 3)	T4	Chemlali	conglomerates, sand and clay	Drop irrigation and use of pomace residue as an amendment
		T4A	Chemlali		
		T4A’	Chemlali		
	Sfax (*n* = 2)	T5	Chemlali	Recent alluvium	nt
		T5A	Chemlali		
	Kasserine (*n* = 2)	T6	Arbequina	sandstone and marl	nt
		T12	ns	limestones, dolostones, marls and gypsum (Zn)	
	Nabeul (*n* = 1)	T7	Zarrazi	ancient limestone and gypsum alluvium	nt
	Kairouan (*n* = 4)	T8	Chemlali	ancient limestone and gypsum alluvium	nt
		T8A	Chemlali		
		T9	Chemlali	Recent alluvium	Drop irrigation
		T9A	Chemlali		
	Jendouba (*n* = 2)	T10	Chetoui	clay-sandstone flysch	nt
		T16	Chetoui	clay-sandstone flysch (Zn and Pb)	
	Ariana (*n* = 3)	T11	Mixture of different varieties	Recent alluvium	nt
		T11A	Chemlali		
		T11A’	Chemlali		
	Beja (*n* = 1)	T13	ns	ancient limestone and gypsum alluvium	nt
	Monastir (*n* = 1)	T14	Mixture of different varieties	conglomerate, sands and clays	nt
	Tozeur (*n* = 1)	T15	Mixture of different varieties	conglomerates, sand and clay	nt
	Siliana (*n* = 1)	T17A	ns	Recent alluvium	nt
Spain (Basque country) (*n* = 7)	Moreda Araba (*n* = 1)	B1	A mixture of Arroniz and Arbequina	ns	Use of fertilizer (15-15-15 NPK *; sheep and cattle manure)
	Añorbe (*n* = 1)	B2	A mixture of Arroniz and Arbequina	ns	ns
	Mendavia (*n* = 1)	B3	Arbequina	ns	ns
	Cintruenigo (*n* = 1)	B4	Arbequina	ns	ns
	Lantziego (*n* = 1)	B5	A mixture of Arroniz and Arbequina	ns	Use of fertilizer (15-15-15 NPK; sheep and cattle manure)
	Ablitas (*n* = 1)	B6	Arroniz	ns	ns
	Oion (*n* = 1)	B7	A mixture of Arroniz and Arbequina	ns	Use of fertilizer (15-15-15 NPK; sheep and cattle manure)
France (*n* = 10)	Nyons (*n* = 2)	FR1	ns	ns	ns
		FR3	ns		
	Baux-de-Provence (*n* = 1)	FR2	Mixture of different varieties	ns	ns
	Nîmes (*n* = 2)	FR4	ns	ns	ns
		FR7	ns		
	Nice (*n* = 2)	FR5	Cailletier	ns	ns
		FR6	ns		
	Provence (*n* = 3)	FR8	Mixture of different varieties	ns	ns
		FR9	Aglandau		
		FR10	ns		

nt: no treatment; ns: not specified, * 15-15-15 NPK: a fertilizer containing equal parts of Nitrogen, Phosphorous, and potassium.

**Table 3:** Measured concentrations of certified and non-certified elements in NIST SRM 2387 peanut butter and recoveries obtained for certified elements.

	Elements	Measured Concentrations ± SD (mg kg^−1^)	Certified Concentrations ± SD (mg kg^−1^)	R (%)
Certified elements (*n* = 3)	Ca	421 ± 6	411 ± 18	102
	Cu	4.26 ± 0.06	4.93 ± 0.15	86
	Fe	16.6 ± 0.3	16.4 ± 0.8	101
	K	6130 ± 160	6070 ± 200	101
	Mg	1696 ± 13	1680 ± 70	101
	Mn	15.5 ± 0.2	16.0 ± 0.6	97
	Zn	25.1 ± 0.3	26.3 ± 1.1	96
Non-certified elements (*n* = 15)	As	0.13 ± 0.07	-	-
	Ba	1.43 ± 0.13	-	-
	Cd	0.051 ± 0.002	-	-
	Co	0.024 ± 0.002	-	-
	Cr	<LOQ	-	-
	Ni	0.78 ± 0.04	-	-
	Pb	<LOQ	-	-
	Rb	5.66 ± 0.23	-	-
	Sr	2.95 ± 0.10	-	-
	V	<LOQ	-	-

**Table 5:** Pearson correlation coefficients and significance between soil and pared olive oil elemental content.

Element	Pearson Correlation Coefficient (r)	Significance (*p*-Value)
Sr	0.85	0.006
Ni	−0.8	0.04
Mg	0.78	0.02
Mn	0.63	0.01
As	−0.12	0.76
Ba	−0.13	0.75
Ca	−0.08	0.85
Cd	nd	nd
Co	−0.42	0.29
Cr	0.03	0.93
Cu	−0.25	0.54
Fe	0.02	0.94
K	−0.32	0.44
Pb	0.08	0.84
Rb	0.57	0.13
V	−0.56	0.14
Zn	−0.42	0.29

nd: not detected.

## Data Availability

All the data are presentd in the paper and in the supplementary material.

## Supplementary Materials

The following supporting information can be downloaded at https://www.mdpi.com/article/10.3390/foods11010082/s1. Table S1. ICP-MS operating conditions and measured isotopes; Table S2. Limits of detection (LOD). Limits of quantification (LOQ), linearity (R2), and precision (RSD); Table S3. Median values and ranges of concentrations (mg kg^−1^) of trace elements in soil extracts from Tunisia.
