# Supplementary material for: Trace Elements Analysis of Tunisian and European Extra Virgin Olive Oils by ICP-MS and Chemometrics for Geographical Discrimination
Source: Foods. 2021 Dec 29;11(1):82. doi: 10.3390/foods11010082 (PMC8750534; doi:10.3390/foods11010082)
Supplement: Supplementary file 1 [file foods-11-00082-s001.zip › foods-1519021-supplementary.pdf]

Supplementary material

Table S1. ICP-MS operating conditions and measured isotopes.

Component / Parameter	Specification / Value
Plasma gas flow	9.0 L min^{-1}
Auxiliary gas flow	1.35 L min^{-1}
Nebulizer gas flow	1.11 L min^{-1}
Spray chamber temperature	3 °C
RF power	1400 W
Sampling depth	5.5 mm
Dwell time	30 ms
Scans per replicate	20
Number replicates per sample	3
iCRC gas (flow)	He, H_2 (120 mL min^{-1})
Isotopes detected, mode “No Gas”	^{24}Mg, ^{85}Rb, ^{88}Sr, ^{114}Cd, ^{137}Ba, ^{208}Pb, ^{89}Y, ^{103}Rh and ^{193}Ir
Isotopes detected, mode “iCRC, He”	^{51}V, ^{52}Cr, ^{55}Mn, ^{59}Co, ^{60}Ni, ^{63}Cu, ^{65}Cu, ^{66}Zn, ^{67}Zn, ^{68}Zn, ^{89}Y, ^{103}Rh and ^{193}Ir
Isotopes detected, mode “iCRC, H_2”	^{39}K, ^{44}Ca, ^{56}Fe, ^{57}Fe, ^{75}As, ^{56}Fe, ^{89}Y, ^{103}Rh and ^{193}Ir

Table S2. Limits of detection (LOD). limits of quantification (LOQ), linearity (R^2) and precision (RSD).

Elements	LOD (μg kg^{-1})	LOQ (μg kg^{-1})	Linearity R^2	RSD (%)
As	0.004	0.014	0.9997	9–11
Ba	0.0003	0.0009	1.0000	4–9
Ca	0.227	0.758	0.9998	2–4
Cd	0.0002	0.0007	1.0000	–
Co	0.001	0.002	1.0000	1–2
Cu	0.006	0.019	0.9999	9–12
Cr	0.035	0.116	1.0000	10–14
Fe	0.015	0.049	0.9997	4–9
K	0.313	1.042	0.9999	1–2
Mn	0.010	0.033	1.0000	7–8
Ni	0.003	0.008	0.9998	13–15
Mg	0.176	0.588	0.9996	2–6
Rb	0.004	0.012	1.0000	1–3
Sr	0.001	0.004	1.0000	10–14
Pb	0.002	0.006	1.0000	1–2
V	0.011	0.037	1.0000	10–15
Zn	0.140	0.468	0.9999	6–8

Table S3. Median values and ranges of concentrations (mg kg^{-1}) of trace elements in soils extracts from Tunisia.

Element	Median value	Minimal – Maximal concentrations
Cd	0.01	[0.003–0.02]
Pb	0.01	[0.002–0.14]
Co	0.04	[0.02–0.13]
Cu	0.07	[0.0004–0.64]
As	0.07	[0.01–0.25]
Cr	0.14	[0.03–0.3]
V	0.15	[0.02–0.59]
Fe	0.25	[0.04–117]
Ni	0.25	[0.08–0.51]
Rb	0.39	[0.17 -0 .84]
Zn	1.86	[0.3–27.7]
Mn	3.23	[1.23–9.49]
Ba	21.5	[7.6–91.5]
Sr	25.6	[11.4–81.4]
Mg	191	[104–772]
K	194	[51.1–551]
Ca	4580	[3140–5990]
